# Interferon-α exacerbates neuropsychiatric phenotypes in lupus-prone mice

**DOI:** 10.1186/s13075-019-1985-9

**Published:** 2019-09-03

**Authors:** Jing Zeng, Xinyu Meng, Ping Zhou, Zhihua Yin, Qinglian Xie, Hong Zou, Nan Shen, Zhizhong Ye, Yuanjia Tang

**Affiliations:** 1 Shanghai Institute of Rheumatology, Renji Hospital, Shanghai Jiao Tong University School of Medicine, 145 Shan Dong Road (c), Shanghai, China; 2 Shenzhen Futian Hospital for Rheumatic Diseases, 22 Nong Lin Road, Shenzhen, China; 3 Shanghai Institutes for Biological Sciences (SIBS), Chinese Academy of Sciences (CAS), University of Chinese Academy of Sciences, 320 Yueyang Road, Shanghai, China; 4 State Key Laboratory of Oncogenes and Related Genes, Shanghai Cancer Institute, Renji Hospital, 2200 Lane 25 Xietu Road, Shanghai, China; 5 Collaborative Innovation Center for Translational Medicine, Shanghai Jiao Tong University School of Medicine, 197 Rui Jin Er Road, Shanghai, China; 6 Center for Autoimmune Genomics and Etiology (CAGE), Cincinnati Children’s Hospital Medical Center, 3333 Burnet Avenue, Cincinnati, OH USA

**Keywords:** Lupus, Autoimmunity, Cytokines, Neuropsychiatric SLE (NP-SLE)

## Abstract

**Background:**

Neuropsychiatric systemic lupus erythaematosus (NP-SLE) is one of the major manifestations of lupus. However, the mechanisms involved in NP-SLE are still largely unknown. The abnormal activation of the type I IFN signalling pathway is involved in SLE pathogenesis and is linked to NP-SLE, but the effect of IFN-α on NP-SLE encephalopathy has not been systematically studied.

**Methods:**

An intravenous injection of Adv-IFN-α (10 mice, 10 × 10^9^ vp) was administered to the IFN-α-treated group, and Adv-ctrl (10 mice, 10 × 10^9^ vp) (ViGene Biosciences, China) was administered to the control group. Gene expression was determined by real-time quantitative polymerase chain reaction (RT-qPCR). Enzyme-linked immunosorbent assay (ELISA) was used to detect antibodies in the serum, and urinary protein levels were measured with a BCA Protein Assay kit. Haematoxylin-eosin (H&E) and periodic acid-Schiff (PAS)-light green staining were used for kidney histology. The elevated plus-maze test, novelty-suppressed feeding assay, open-field test, tail suspension test, social dominance tube test, three-chamber social interaction test, step-down passive avoidance test and novelty Y-maze task were used to assess behaviour.

**Results:**

In this study, we performed a series of behavioural tests to assess the neuropsychiatric phenotypes of IFN-α-treated NZB/NZW F1 mice and found that these mice developed a series of mental disorders such as anxiety-like phenotypes, depression-like phenotypes, deficits in sociability and cognitive impairments, which mimic the neuropsychiatric manifestations of NP-SLE, with a consistent onset and progression.

**Conclusions:**

Our research verified that IFN-α plays a critical role in NP-SLE and provides a comprehensive NP-SLE mouse model for dissecting the mechanisms of NP-SLE and developing novel therapies for intervention.

**Electronic supplementary material:**

The online version of this article (10.1186/s13075-019-1985-9) contains supplementary material, which is available to authorized users.

## Introduction

Systemic lupus erythaematosus (SLE) is a complex systemic autoimmune disease characterized by a loss of immune tolerance to self-antigens, resulting in inflammation and severe end-organ damage. Almost all organ systems can be affected, with disease manifestations and severity displaying heterogeneity within and between patients and ranging from the common involvement of skin and joints to life-threatening renal or neuropsychiatric SLE (NP-SLE) [1, 2]. As one of the most debilitating and potentially fatal manifestations of lupus, NP-SLE is associated with poor prognosis, more cumulative organ damage, anxiety, depression, sociability deficits, cognitive impairment and seizures [3–5].

Despite years of study, the aetiology of NP-SLE is still largely unclear. The abnormal activation of the type I interferon (IFN) pathway has received particular attention for its role in the pathogenic mechanisms underlying SLE; the role of this pathway was initially demonstrated by the induction of a signature of IFN inducible genes in human monocytes exposed to SLE sera [6] and then further corroborated by gene expression microarray studies of peripheral blood cells in SLE [7–10]. Meanwhile, the abnormal activation of the type I interferon (IFN) pathway has also been demonstrated to be closely related to NP-SLE onset and development. The connection between IFN-α and human mental disorders was first identified in patients who frequently exhibited neurotoxicity and neuropsychiatric side effects such as anxiety, depression, irritability, a lack of motivation and impaired memory after receiving a high dose of IFN-α [11, 12]. Similar mental disorders have also been observed in healthy volunteers and mice peripherally administered IFN-α [12–14], and abnormal levels of IFN-α in the sera and cerebrospinal fluid (CSF) of patients have been observed [15, 16], supporting the direct interaction between IFN-α and behavioural symptoms. However, the effect of IFN-α on the overall phenotype of NP-SLE encephalopathy remains undefined.

In efforts to explore the potential mechanisms and enhance the development of novel therapeutics, numerous mouse models of SLE, including genetically predisposed mouse strains, such as the NZB/NZW F1 strain and its NZM derivatives, the MRL/lpr strain and the lupus-prone 564Igi strain, as well as inducible systems and humanized mouse models of lupus, have been used [17–20]. These models share lupus-like phenotypes including proteinuria, elevated serum dsDNA antibodies, antinuclear antibodies (ANAs), anti-ribosomal P antibodies (anti-P) to immunoglobulin G (IgG)-containing immune complex deposits in the kidneys and renal failure. However, in terms of NP-SLE, none of these models exhibit all of the clinical symptoms found in SLE patients. For example, the 564Igi strain exhibits anxiety-like behaviour and motor and coordination defects but lacks a depression-like phenotype [20]; the MRL/lpr strain exhibits depression-like behaviour and deficits in cognitive function without anxiety-like behaviour [21]; and NZB/NZW F1 mice exhibit congenital structural abnormalities of the brain and mental disorders such as increased anxiety-like behaviours and decreased locomotor activity [22]. The NZB/NZW F1 mouse model, which relies on inbred mouse strains, exhibits delayed and inconsistent disease onset, with SLE-related symptoms usually not appearing until 5–6 months of age; this remains a distinct limitation of this classic model. Recently, an injection of an adenovirus expressing IFN-α (Adv-IFN-α) in 12- to 13-week-old NZB/W F1 mice was found to accelerate the main clinical symptoms of SLE, making IFN-α-accelerated NZB/NZW F1 mouse models a useful tool to explore the intricate pathogenesis of SLE [23]. To date, studies on the IFN-α-accelerated NZB/NZW F1 model have provided many new target molecules for SLE, such as miR-130b as a negative regulator in the IFN pathway [24], a Btk inhibitor as a B cell regulator [25] and Ox40 as a molecule involved in a novel pathogenic pathway of SLE [26]. However, no study has revealed the association between the IFN-α-accelerated NZB/NZW F1 mouse model and NP-SLE.

In the present study, we characterized the behavioural profile of the IFN-α-accelerated NZB/NZW F1 mouse model. Compared with the control group, IFN-α-treated lupus-prone mice exhibited more serious mental disorders, including anxiety-like phenotypes, depression-like phenotypes, decreased aggression, abnormal social interaction and impaired cognitive performance and long-term memory, which mimic the overall phenotypes of NP-SLE encephalopathy. Our findings demonstrated that IFN-α-accelerated NZB/NZW F1 mice can serve as a comprehensive NP-SLE mouse model.

## Methods

### Animals

Female NZB/NZW F1 mice (strain 100,008) aged 12–13 weeks and weighing 30 ± 2 g were purchased from the Jackson Laboratory and maintained at the Shanghai Institutes for Biological Sciences, Chinese Academy of Sciences. The mice were kept in a regulated environment (23 ± 1 °C, 40 ± 60% humidity) under a 12-h light/12-h dark cycle and free access to laboratory chow and water. The mice were treated at the age of 12 weeks with a single intravenous injection of Adv-IFN-α (10 mice; 10 × 10^9^ vp) for the IFN-α-treated group or Adv-ctrl (10 mice; 10 × 10^9^ vp) (ViGene Biosciences, China) for the control group, as previously described [23]. Urine was tested weekly for proteinuria by the BCA assay. Behavioural experiments were performed when the mice were 20 weeks of age. The levels of anti-dsDNA, ANAs and anti-P antibodies in the serum were measured by ELISA. All of the surviving mice were sacrificed at 24 weeks of age. All animal protocols were approved by the Animal Care and Use Committee of the Renji Hospital.

### Proteinuria measurement

The mice were housed in metabolic cages, and their urine was collected over a 24-h period. Urinary protein was measured with a BCA Protein Assay kit according to the manufacturer’s instructions (Tiangen Biotech). The samples were measured in duplicate with three to 10 animals per group.

### ELISA

Serum was obtained from 24-week-old mice, and the levels of anti-dsDNA, ANAs and anti-P antibodies were measured with anti-dsDNA, ANA and anti-P ELISA kits (Inova Diagnosis) according to the manufacturer’s instructions. The samples were measured in duplicate with 3 to 10 animals per group.

### Real-time qPCR

Total RNA was isolated using TRIzol reagent (Invitrogen). Gene expression was assayed using TB Green Premix Ex Taq reagent (TAKARA) according to the manufacturer’s protocol and normalized to GAPDH mRNA levels. All experiments were assayed on an ABI ViiA 7 Real-Time PCR System (Applied Biosystems). All primers for SYBR Green real-time qPCR were synthesized by Sangon Biotech. The primers are as follows: mmu-IFN-α-forward (5′-3′), ATG GCT AGG CCC TTT GCT TTC; mmu-IFN-α-reverse, CTG TGT ACC AGA GGG TGT AGT T; mmu-CXCL10-forward, GAC GGT CCG CTG CAA CTG; mmu-CXCL10-reverse, GCT TCC CTA TGG CCC TCA TT; mmu-IL-6-forward, CCA GTT GCC TTC TTG GGA CT; mmu-IL-6-reverse, GTC TCC TCT CCG GAC TTG TG; mmu-GAPDH-forward, CAG AAC ATC ATC CCT GCA TC; and mmu-GAPDH-reverse, CTG CTT CAC CAC CTT CTT GA.

### Kidney histology

Kidneys were fixed in formalin and embedded in paraffin. The blocks were sectioned at 2.5 μm. Two kidney sections were prepared. One was stained with haematoxylin-eosin (H&E), and the other was stained with periodic acid-Schiff (PAS)-light green staining. The slides were identified with the animal number and then examined under a microscope at × 400 magnification (Nikon, Japan) by a researcher blinded to treatment and proteinuria levels.

### Behavioural experiments

All behavioural experiments were performed between 9:00 and 17:00 in a soundproof and air-regulated experimental room, to which the mice were habituated at least 30 min before each test. There was at least 1 day between each behavioural test and the next. The mice were tested in random order. Following testing, the apparatus was cleaned with 70% ethanol and water to remove olfactory traces.

### Elevated plus-maze test

The elevated plus-maze test is used to evaluate anxiety-like phenotypes [27]. The apparatus consisted of two open Plexiglas arms (l × w, 78 cm × 5 cm) and two enclosed Plexiglas arms (l × w × h, 78 cm × 5 cm × 20 cm). The arms of the same type were located opposite one another and extended from a central platform (l × w, 7.5 cm × 7.5 cm), and the maze was elevated to a height of 60 cm above the floor. The test was carried out in dim ambient lighting (~ 5 lx) for 5 min. The test mouse was placed in the centre of the platform facing an open arm and was allowed to explore. In this test, when all four paws of the mouse were in an arm, an entry was recorded. Activity in the open arm was used to represent anxiety-like behaviour. The total distance travelled was used as a measure of general locomotor activity.

### Novelty-suppressed feeding assay

The novelty suppressed feeding assay is a conflict test that elicits competing motivations between the drive to eat and the fear of venturing into the centre of a brightly lit arena [28]. The apparatus consisted of a square base (l × w: 60 cm × 60 cm) with a small pre-weighed food chow pellet (normal chow) placed in the centre of the arena on a small fixed glass dish. During the test, the brightness level of the lights above the apparatus was increased to the highest level (~ 35 lx). The mice were individually placed in a clean cage without food 18 h before testing. Feeding was defined as the mouse grabbing the pellet and starting to eat. The mouse was removed after 10 min and then placed alone in its home cage for 5 min with another pre-weighed piece of chow. At the end of the 5-min period, the amount of food consumed was also measured. The latency for a mouse to approach and eat a familiar food in a novel environment following an extended period (16–24 h) of food deprivation was used as an index of anxiety-like behaviour. Food consumption was used to test whether the experimental condition altered appetite or motivation to eat.

### Open-field test

The open-field test was used to assess the locomotor and exploratory activity of the mice when they were placed in an unfamiliar environment [29, 30]. The open-field chambers (l × w × h, 60 cm × 60 cm × 60 cm) were made of Plexiglas with a non-reflective square base, and the centre area (30 cm × 30 cm) was marked. The area between the background and 80% of the centre area (48 cm × 48 cm) was defined as the margin area. For each testing session, a mouse was placed individually in the centre of the arena and allowed to freely explore the environment for 30 min. The total distance travelled (mm) was used as a measurement of general locomotor activity.

### Tail suspension test

The tail suspension test was used to establish a despair and helpless situation to test depression-like behaviours in mice [31, 32]. At the start of the experiment, a mouse was suspended by its tail 90 cm above the floor. During the test (6-min trial), the behaviour of the mouse was recorded, and any period in which the mouse stopped struggling for ≥ 1 s was considered freezing. The latency to freezing and the total freezing time in the last 4 min were measured manually in a blinded manner.

### Social dominance tube test

The social dominance tube test was used to measure the dominance of animals [33]. The test was conducted in a testing tube (l × internal diameter, 30.5 cm × 2.5 cm), through which only one mouse can pass. Sex-matched and weight-matched test mice that were unfamiliar with each other were used. Before the test trials, all mice were adapted to the testing tube by being guided across the testing tube in alternating directions. All mice were confirmed to be free to pass through the tube. After training and habituation, the mice were randomly placed at opposite ends of the test tube and released at the same time so that the mice were able to meet in the middle of the tube. Ten consecutive trials were performed with a maximum time of 3 min per trial. During the trial, if one mouse backed out of the tube and all four of its paws were outside of the tube, the trial ended, and that mouse lost. Matches lasting more than 3 min were scored as a draw and excluded from the analysis. Mice were considered socially dominant if they won more than 50% of the trials.

### Three-chamber social interaction test

The three-chamber social interaction test was used to assess sociability and preference for social novelty [33, 34]. The test was performed in a rectangular apparatus made of white Plexiglas (l × w × h, 66 cm × 40 cm × 24 cm) that contained three equally sized chambers with a gate (l × w, 6 cm × 4 cm) between the chambers. The right and left chambers each had a wire cage that was 11 cm in height and 9 cm in diameter. The test was started by placing a mouse into one of the cages. The following behaviours were scored: the amount of time spent and the distance travelled in each chamber of the three-chamber arena and the amount of time spent and the distance travelled in close proximity (within 3 cm) of the wire cage.

### Step-down passive avoidance test

The step-down passive avoidance test was used to assess the learning and memory capacity of the mice [35]. The test was performed in a Plexiglas cage (l × w × h, 20 cm × 20 cm × 20 cm) with a small platform (l × w × h, 8 cm × 8 cm × 1.5 cm) in the corner. The experiment began with a training session in which each mouse was placed on the platform. When the mouse stepped down on the grid door with its four paws, footshocks (50 V) lasting 5 s were delivered to the grid floor. The mouse was again placed on the platform. If the mouse stayed on the platform for at least 3 min, it was considered to remember the shock. If the mouse failed to stay on the platform for at least 3 min for 2 times, it was excluded. Two hours and 24 h later, the mouse was placed on the platform again. The latency to step down with a cutoff of 10 min was measured. The shocks were not delivered to the grid door during the two sessions in which the mouse stepped down. The following two parametric measures of retention were measured manually in a blinded manner: the latency to step down and the number of mice that reached the avoidance criterion.

### Novelty Y-maze task

The novelty Y-maze task was used to assess short-time spatial recognition working memory in mice by measuring spontaneous alternations [36, 37]. The maze consisted of three arms (one starting arm and two testing arms); each arm was 31 cm long, 5 cm wide and 10 cm high with a central area in the shape of an equilateral triangular. Each arm had markers of different colours as distinct visual cues and a removable partition to block the appropriate arm. The test consisted of two sessions, and at the beginning of each session, the test mouse was placed in the same starting arm. However, in the first session, only the starting arm and one testing arm (also known as familiar arm (fa)) were open, while the second testing arm (also known as novel arm (na)) was blocked by the partition. The blocked arm was randomized and balanced for each test mouse. The test mouse was placed in the starting arm and allowed to explore the open testing arm for 5 min. The mouse was then returned to its home cage for a 30-min interval. After the maze was cleaned and the partition was removed, the test mouse was placed in the starting arm again and allowed to explore the two testing arms for another 5 min. For each test mouse, the percentage of time spent exploring and distance travelled in the novel arm during the second session was calculated using the following formula: percentage of time/distance = time or distance in na/(time or distance in na + time or distance in fa) × 100.

### Statistical analyses

We used TopScan Lite software (CleverSys, Inc.) to record, track and analyse the tests. In the elevated plus-maze test, the number of arm entries as well as the total time spent and distance travelled in the open, closed and centre compartments was analysed; in the novelty-suppressed feeding assay, the latency to the first feeding event for each mouse was analysed; in the open-field test, mouse behaviour during the test period was analysed; and in the three-chamber social interaction test, the performance of each mouse was analysed. GraphPad Prism 7 software (Graph-Pad Software, Inc.) was used for data analysis.

## Results

### IFN-α treatment induced autoimmune status in NZB/NZW F1 mice

As a complex and common autoimmune disease, SLE is characterized by highly diverse clinical manifestations, and the prevalent IFN signature is considered one of the most robust biomarkers. Previous studies have found that IFN-α treatment promotes SLE pathogenesis in NZB/NZW F1 mice.

**Fig. 1:**
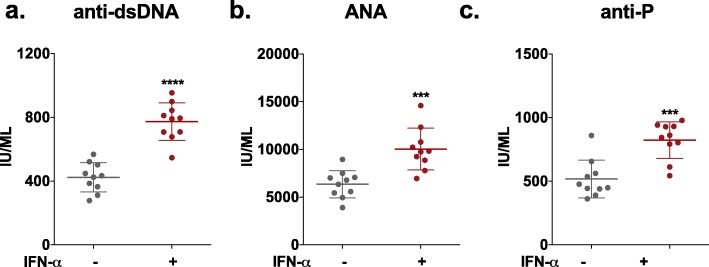
Effect of IFN-α on antibodies in NZB/NZW F1 mice. **a** Serum levels of antibodies reactive to double-stranded DNA (dsDNA), as detected by ELISA. **b** Serum levels of antibodies reactive to ANAs, as detected by ELISA. **c** Serum levels of antibodies reactive to anti-P, as detected by ELISA. Number of animals per group = 10. *P < 0.05, **P < 0.01, ***P < 0.005, ****P < 0.001 vs. Adv-ctrl-treated group, unpaired *t* test

Consistent with the literature, in our study, we observed that IFN-α-treated mice exhibited significantly higher levels of anti-dsDNA and ANAs (Fig. 1a, b) as well as increased proteinuria (Additional file 1: Figure S1a) compared to those in control mice (Adv-ctrl-treated mice). Haematoxylin-eosin (H&E) and periodic acid-Schiff (PAS) staining were performed to assess global kidney morphology (Additional file 1: Figure S1b), and more serious pathological changes of the renal glomerulus were observed in IFN-α-treated mice. In addition, IFN-α treatment resulted in significantly higher levels of anti-P (Fig. 1c), which is associated with SLE-related psychiatric and nephritic manifestations [38]. In addition, real-time qPCR analysis also showed the upregulation of IFN-α, IL-6 and CXCL10, which are factors associated with inflammation of the brain [39–42], in the brain tissue of IFN-α-treated NZB/NZW mice compared to control mice (Additional file 2: Figure S2).

The abovementioned results all confirm that the injection of Adv-IFN-α in NZB/NZW F1 mice accelerated the main clinical symptoms of SLE and resulted in corresponding inflammation in the brain and kidneys of mice, suggesting that it activated autoimmune status. To further verify whether the mice had corresponding behavioural changes, we conducted a series of subsequent investigations.

### Increased anxiety-like phenotypes in IFN-α-treated NZB/NZW F1 mice

Anxiety is one of the main mental symptoms of the neuropsychiatric manifestations of lupus. To assess whether the IFN-α-treated NZB/NZW F1 mouse model can serve as a model of NP-SLE, we performed the elevated plus-maze test, the novelty-suppressed feeding assay and the open-field test to examine anxiety-related behavioural phenotypes.

**Fig. 2:**
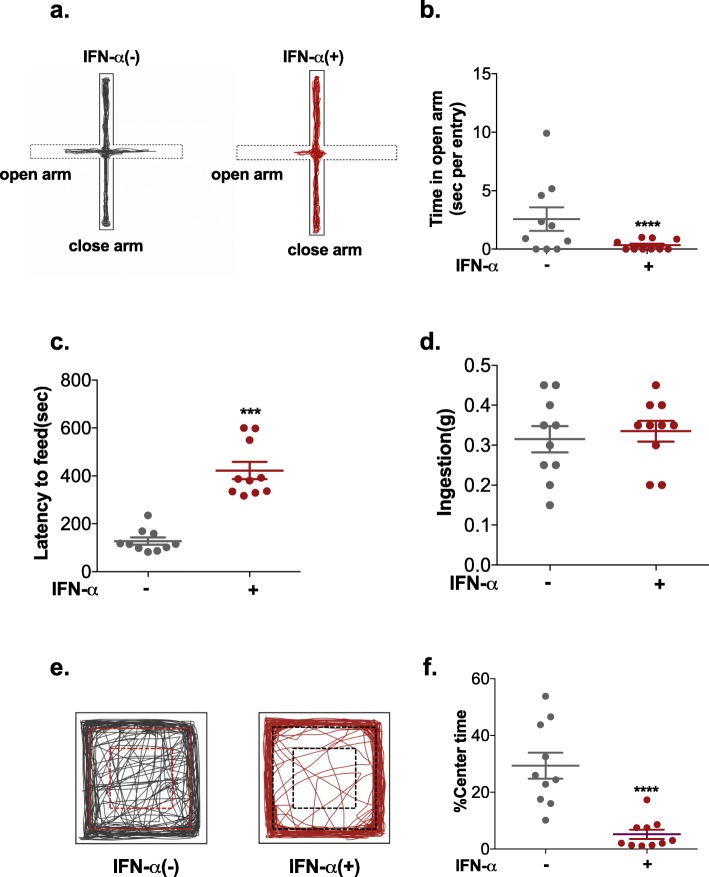
Effect of IFN-α on anxiety-like phenotypes in NZB/NZW F1 mice, as assessed by the elevated plus-maze test, novelty-suppressed feeding assay and open-field test. **a** Representative activity traces of a control mouse and an IFN-α-treated mouse in the elevated plus maze-test. **b** Time spent in the open arms per entry. Latency to feed (**c**) and food consumption (**d**) of the mice in the novelty-suppressed feeding assay**. e** Illustrative example of the travel path of a control mouse and an IFN-α-treated mouse in the open field. **f** Percentage of time spent in the centre of the open-field arena. Number of animals per group = 10. *P < 0.05, **P < 0.01, ***P < 0.005, ****P < 0.001 vs. Adv-ctrl-treated group, unpaired *t* test

In the elevated plus-maze test, the level of anxiety-like phenotypes was measured based on the time spent in and the number of entries into the open arms. Less time spent in and fewer entries into the open arms indicated anxiety [27]. Compared to control mice, IFN-α-treated mice spent significantly less time and travelled a shorter distance in the open arms (Fig. 2b, Additional file 3: Figure S3b). The frequency of entry into the open arms (Additional file 3: Figure S3d) was also strongly reduced in the IFN-α-treated mice group, but there was no significant difference in the total distance travelled in (Additional file 3: Figure S3a) or the number of entries (Additional file 3: Figure S3c) into either the open or closed arms. These results suggested that IFN-α-treated mice avoided the aversive open arms of the maze.

In the novelty-suppressed feeding assay, the latency of each mouse to approach and eat a familiar food was used as an index of anxiety-like behaviour with longer latency indicating anxiety [43]. As shown in Fig. 2c, the latency to feeding increased sharply in IFN-α-treated mice. Moreover, no differences in food consumption in the home cage were observed among the groups (Fig. 2d).

In the open-field test, no significant differences were observed in the total distance travelled between the two groups (Additional file 3: Figure S3e), indicating that IFN-α treatment had no influence on locomotor activity. In the open-field test, the time spent in, number of entries into and distance travelled in the centre area and margin area are indices of anxiety-like behaviours in mice. Compared with control mice, IFN-α-treated mice travelled a longer distance in the margin area (Additional file 3: Figure S3f) and spent less time in (Fig. 2f) and made fewer entries into the centre area (Additional file 3: Figure S3 g), suggesting anxiety-like behaviour.

Taken together, our data demonstrate that IFN-α treatment resulted in increased anxiety-like phenotypes in NZB/NZW F1 mice, which is similar to NP-SLE.

### IFN-α treatment induced depression-like behaviours in NZB/NZW F1 mice

**Fig. 3:**
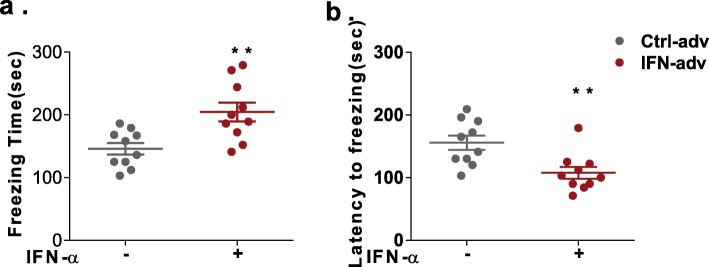
Effect of IFN-α on depressive-like behaviours in NZB/NZW F1 mice, as assessed by the tail suspension test. The freezing time (**a**) and latency to freezing (**b**) of the mice in the tail suspension test. Number of animals per group = 10. *P < 0.05, **P < 0.01, ***P < 0.005, ****P < 0.001 vs. Adv-ctrl-treated group, unpaired *t* test

To further evaluate the NP-SLE phenotypes of this model, we monitored depression-like behaviours in NZB/NZW F1 mice with the tail suspension test. The freezing duration and the latency to freezing were used as measures of depression-like behaviours [31]. Compared with control mice, IFN-α-treated mice exhibited a longer duration of freezing during the tail suspension test (Fig. 3a). Similarly, the latency to freezing was also decreased significantly in the IFN-α-treated mice (Fig. 3b). These results reveal increased depressive-like behaviour in IFN-α-treated mice.

### Deficits of sociability in IFN-α-treated NZB/NZW F1 mice

As impairment in social behaviours is also a common symptom of NP-SLE, we employed the social dominance tube test and the three-chamber social test to evaluate the sociability of IFN-α-treated NZB/NZW F1 mice.

**Fig. 4:**
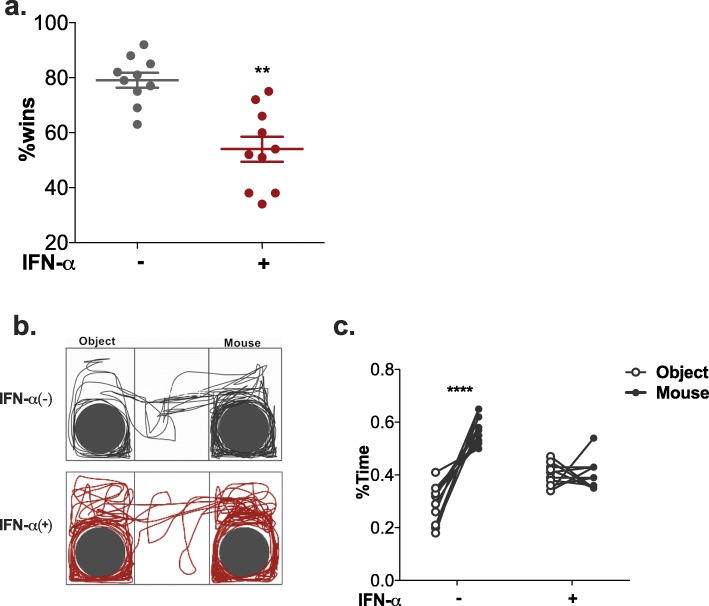
Effect of IFN-α on sociability in NZB/NZW F1 mice, as assessed by the social dominance tube test and the three-chamber social interaction test. **a** Graph showing the percentage of wins in the tube test. Number of animals per group: *n* = 10. *P < 0.05, **P < 0.01, ***P < 0.005, ****P < 0.001 vs. Adv-ctrl-treated group, unpaired *t* test. **b** Illustrative example of the travel path of a control mouse and an IFN-α-treated mouse in the three-chamber interaction test. **c** Percentage of time spent in the two chambers. Two-way ANOVA with Sidak’s test

We first performed the social dominance tube test to evaluate aggression and dominance. Our results showed that the IFN-α-treated mice had a much smaller probability of winning, indicating decreased aggressive tendencies (Fig. 4a).

In the three-chamber social test, social versus empty cage preference was used to reflect the sociability of the mice. No significant differences were observed in the total distance travelled in the three chambers between IFN-α-treated mice and control mice (Additional file 4: Figure S4a), but the chamber in which they chose to stay was obviously different. Based on the amount of time spent (Fig. 4b) and distance travelled (Additional file 4: Figure S4b) in the chambers, control mice showed a preference to stay in the chamber with the cage containing a novel mouse rather than the chamber containing an empty cage. However, IFN-α-treated mice showed no significant difference in the time spent or distance travelled in the two chambers (Fig. 4c, Additional file 4: Figure S4b). To further analyse the direct social approach behaviours, we calculated the percentage of sniffing time and the circling distance around the cages. Based on sniffing time (Additional file 4: Figure S4c) and circling distance (Additional file 4: Figure S4d) around the cages containing a novel mouse than around the empty cage, control mice also showed significant social preference, while the IFN-α-treated mice did not.

Altogether, these results suggest that IFN-α treatment impaired sociability mice.

### Long-term memory and spatial working memory deficits in IFN-α treated NZB/NZW F1 mice

We further evaluate whether IFN-α affects cognitive function in NZB/NZW F1 mice with the step-down passive avoidance test and the novelty Y-maze task.

**Fig. 5:**
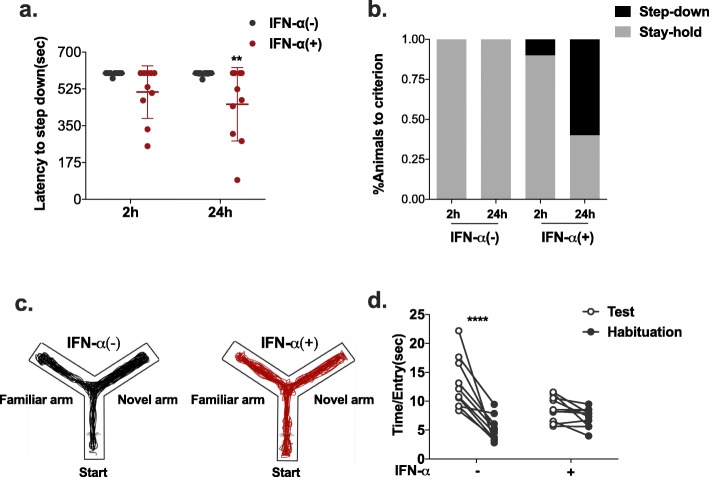
Effect of IFN-α on cognitive function in NZB/NZW F1 mice, as assessed by the step-down passive avoidance test and the novelty Y-maze task. Latency (**a**) and percentage to reach criterion (**b**) of IFN-α-treated mice and control mice in the step-down passive avoidance test and in the novelty Y-maze task. **c** Illustrative example of the travel path of a control mouse and an IFN-α-treated mouse in the Y-maze. **d** Time spent in the arms per entry. Number of animals per group = 10. *P < 0.05, **P < 0.01, ***P < 0.005, ****P < 0.001 vs. Adv-ctrl-treated group, two-way ANOVA with Sidak’s test

In the step-down passive avoidance test, the latency to step down and the percentage of mice that reached the avoidance criterion were adopted as measures of memory performance [44]. As shown in Fig. 5a, we observed no difference in the latency to step down between the two groups in the short term (2 h) but a much shorter latency in the long term (24 h) than in control mice. As for the percentage of mice to reach criterion (Fig. 5b), IFN-α treatment also exerted a suppressive effect on long-term memory.

The novelty Y-maze task was used to measure the willingness of mice to explore new environments and to assess spatial recognition working memory. Mice typically prefer to investigate the novel arm of the maze rather than returning to one that was visited previously, which is assumed to represent functional working memory [36]. We examined the effect of IFN-α on short-term spatial working memory by monitoring the spontaneous alternation behaviour of mice in the Y-maze. We found little difference between groups in the total distance travelled (Additional file 5: Figure S5a) and the number of arm entries (Additional file 5: Figure S5b), but there was a strong regression of performance after IFN-α treatment, as determined by less time spent and distance travelled in the novel arm (Fig. 5d, Additional file 5: Figure S5c).

These findings suggest that IFN-α treatment strongly interfered with long-term memory recovery and spatial recognition working memory.

## Discussion

Neuropsychiatric systemic lupus erythaematosus (NP-SLE) refers to a series of neurological and psychiatric symptoms directly related to SLE. The functional mechanism of NP-SLE remains largely elusive, and an animal model that accurately mimics the clinical presentation of NP-SLE would be indispensable. In our study, IFN-α-treated mice were found to exhibit rapid and highly synchronized onsets of SLE and psychiatric symptoms such as anxiety, depression, deficits in sociability and cognitive impairment, mimicking the neuropsychiatric manifestations of NP-SLE and providing evidence of the critical role of IFN-α in brain physiology and CNS disorders.

In recent decades, several studies have suggested that the abnormal activation of the IFN pathway participates in NP-SLE onset. By blocking type I interferon (IFN) signalling, IFN-α has been proven to drive microglia-dependent synapse loss, contributing to impaired cognitive function and sociability in lupus-prone mice [20]. The key IFN signalling molecule STAT1 was identified by global gene profiling to be highly elevated and activated in neurons in the central nervous system [45, 46], while the IFN-stimulated gene USP18 (ubiquitin-specific protease 43 (UBP43)) was found to affect brain cell function though the regulation of the cellular levels of the ISG15 protein [47] and CXCL10 (endothelia-derived chemokine ligand)-mediated behavioural changes through the impairment of synaptic plasticity [48]. In addition, a novel and rare variant of the IFN regulatory gene TREX1 identified by whole-exome sequencing following biochemical analysis in CNS-lupus patients has been proven to result in the abnormal increase in IFN-α found in the cerebrospinal fluid (CSF) [49]. Moreover, TREX1 deficiency in IFN-α-dependent T helper 1 (Th1) cells has been found to inhibit endothelial cell (EC) angiogenesis [50]. Altogether, we proved the direct effect of IFN-α on the pathogenesis of NP-SLE, but we are still at an early stage in understanding the overall pathogenesis. It is necessary to determine whether IFN-α is involved in NP-SLE through its effects on the immune system, nervous system or both. The IFN-α-accelerated NZB/NZW F1 mouse model can be used to dissect the cellular and molecular mechanisms of NP-SLE.

The sustained activation of the type I IFN pathway is the main molecular phenotype in lupus patients. In recent studies, IFN-α-adenoviral vectors have been proven to be good candidates for gene transfer in vivo [51] and to induce long-lasting expression of IFN-α [23, 52, 53]. In our study, the production of IFN-α was also found to be sustained in the IFN-α-accelerated NZB/NZW F1 mouse model. Adv-IFN-α treatment synchronized and expedited SLE onset in the IFN-α-accelerated NZB/NZW F1 mouse model. The IFN-α-accelerated NZB/NZW F1 mice also showed manifestations similar to those of NP-SLE, such as anxiety-like phenotypes, depression-like behaviours, deficits in sociability and long-term memory and spatial working memory deficits. In future studies, neuropathological changes such as microglial activation, neuronal and synaptic structure, cerebrospinal fluid (CSF) profile and magnetic resonance imaging (MRI) and changes in the cerebral expression of IFN-regulated genes should be evaluated in the IFN-α-accelerated NZB/NZW F1 mouse model.

## Conclusions

In conclusion, our findings provide evidence that IFN-α directly participates in NP-SLE pathogenesis and results in brain physiology and CNS disorders. In addition, with increased interest in IFN-α in NP-SLE, our study also offers a useful rodent model for research on the cellular and molecular mechanisms underlying NP-SLE.

## Additional files

Additional file 1: **Figure S1.** Effect of IFN-α on lupus nephritis in NZB/NZW F1 mice. (a) Proteinuria, as quantified by 24-h urinary protein. (b) H&E- or PAS-stained kidney sections. Number of animals per group =5. *P < 0.05, **P < 0.01, ***P < 0.005, ****P < 0.001 vs. Adv-ctrl treated group, unpaired *t* test. (PDF 311 kb)

Additional file 2: **Figure S2.** The expression levels of IFN-α, IL-6 and CXCL10 in the brain. Number of animals per group =5. *P < 0.05, **P < 0.01, ***P < 0.005, ****P < 0.001 vs. Adv-ctrl treated group, 2-way ANOVA with Sidak’s test. (PDF 34 kb)

Additional file 3: **Figure S3.** Effect of IFN-α on anxiety-like phenotypes in NZB/NZW F1 mice, as assessed by the elevated plus-maze test. (a) Total distance travelled in the elevated plus-maze. (b) Percentage of distance travelled in the open arm of the elevated plus-maze. (c) Total number of entries into the arms of the elevated plus-maze. (d) Percentage of entries into the open arms. (e) Total distance travelled in the open field arena. (f) Percentage of distance travelled in the margin area in the open field test. (g) Number of entries into the centre of the open field. Number of animals per group =10. *P < 0.05, **P < 0.01, ***P < 0.005, ****P < 0.001 vs. Adv-ctrl treated group, unpaired t test. (PDF 44 kb)

Additional file 4: **Figure S4.** Effect of IFN-α on sociability in NZB/NZW F1 mice, as assessed in the three-chamber social interaction test. (a) Total distance travelled in all of the three chambers. (b) Percentage of distance travelled in the two chambers. Percentage of time spent (c) and distance travelled (d) around the two cages. Number of animals per group =10. *P < 0.05, **P < 0.01, ***P < 0.005, ****P < 0.001 vs. Adv-ctrl treated group, unpaired t test (a), 2-way ANOVA with Sidak’s test (b, c, d). (PDF 55 kb)

Additional file 5: **Figure S5.** Effect of IFN-α on sociability in NZB/NZW F1 mice, as assessed by the novelty Y-maze task. (a) Total distance travelled. (b) Total number of arm entries. (c) Distance travelled in the arms per entry. Number of animals per group =10. *P < 0.05, **P < 0.01, ***P < 0.005, ****P < 0.001 vs. Adv-ctrl treated group, unpaired t test (a, b), 2-way ANOVA with Sidak’s test (c). (PDF 35 kb)

## Data Availability

Not applicable.

## Abbreviations

Adv-IFN-α: Adenovirus expressing IFN-α
ANA: Antinuclear antibody
CNS: Central nervous system
CSF: Cerebrospinal fluid
CXCL10: Chemokine (C-X-C motif) ligand 10
dsDNA: Double-stranded DNA
ECs: Endothelial cells
HE: Haematoxylin-eosin staining
IFN: Type I interferon
IgG: Immunoglobulin G
IL-6: Interleukin-6
MRI: Magnetic resonance imaging
NP-SLE: Neuropsychiatric SLE
P: Ribosomal P
PAS: Periodic acid-Schiff
SLE: Systemic lupus erythaematosus
Th1: T helper 1
