# Supplementary figures and images for: Interferon-α exacerbates neuropsychiatric phenotypes in lupus-prone mice
Source: Arthritis Res Ther. 2019 Sep 3;21:205. doi: 10.1186/s13075-019-1985-9 (PMC6724270; doi:10.1186/s13075-019-1985-9)

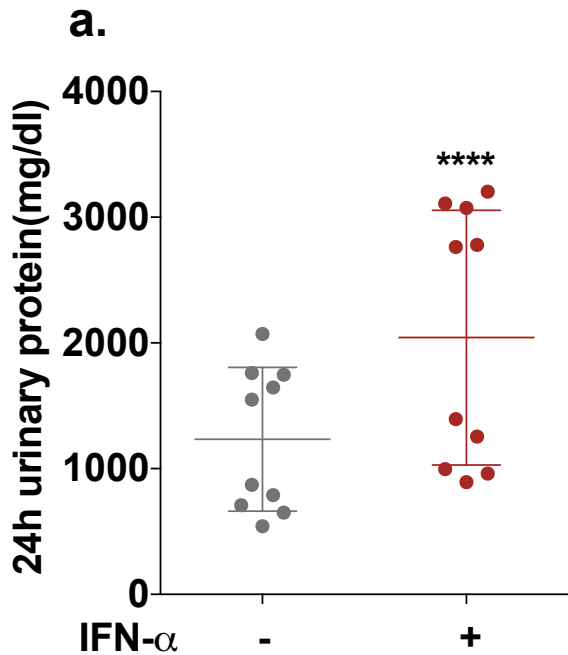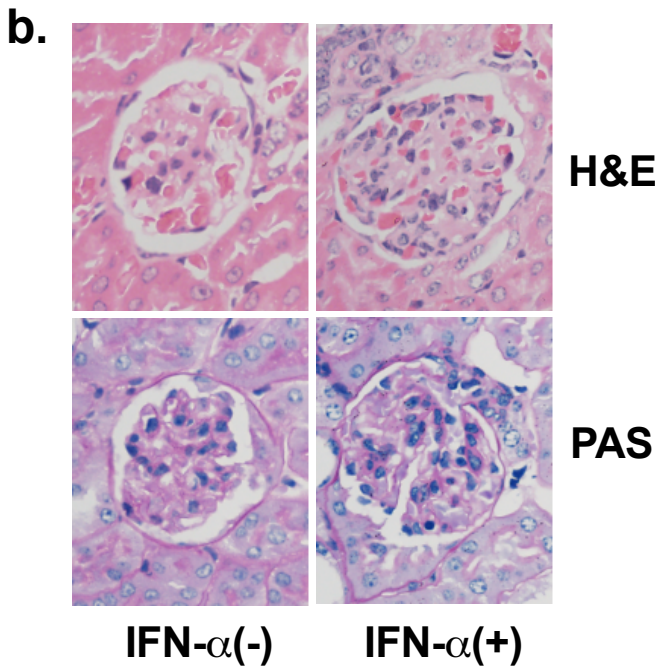

Supplement: Supplementary file 1 — Figure S1. Effect of IFN-α on lupus nephritis in NZB/NZW F1 mice. (a) Proteinuria, as quantified by 24-h urinary protein. (b) H&E- or PAS-stained kidney sections. Number of animals per group =5. *P < 0.05, **P < 0.01, ***P < 0.005, ****P < 0.001 vs. Adv-ctrl treated group, unpaired t test. (PDF 311 kb) [file 13075_2019_1985_MOESM1_ESM.pdf]

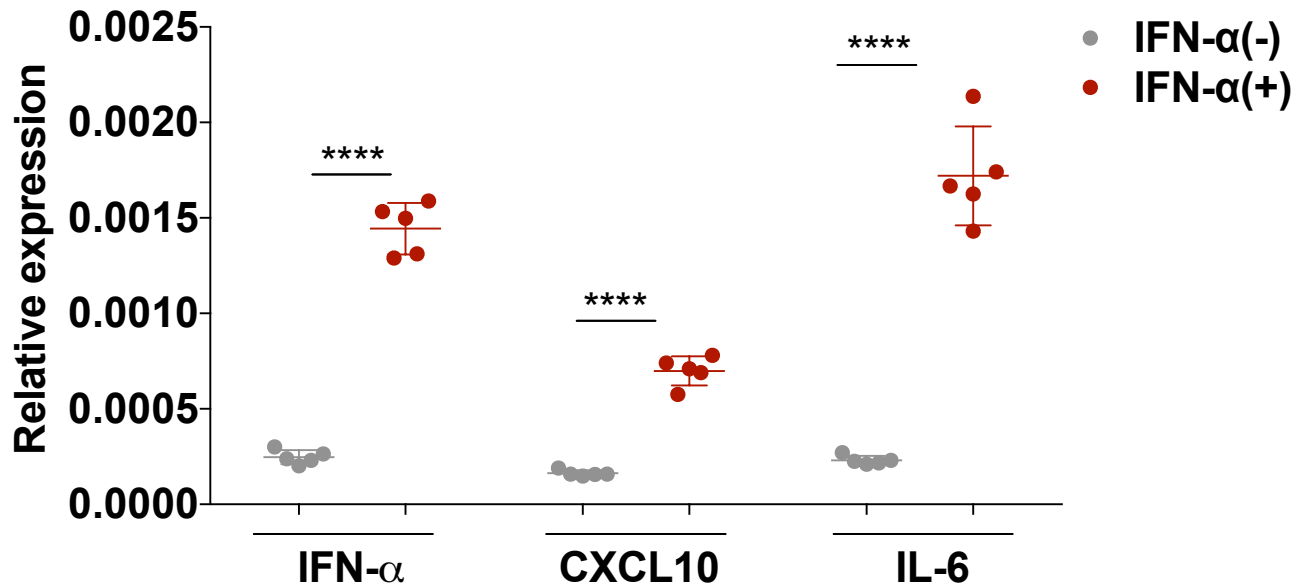

Supplement: Supplementary file 2 — Figure S2. The expression levels of IFN-α, IL-6 and CXCL10 in the brain. Number of animals per group =5. *P < 0.05, **P < 0.01, ***P < 0.005, ****P < 0.001 vs. Adv-ctrl treated group, 2-way ANOVA with Sidak’s test. (PDF 34 kb) [file 13075_2019_1985_MOESM2_ESM.pdf]

**a.**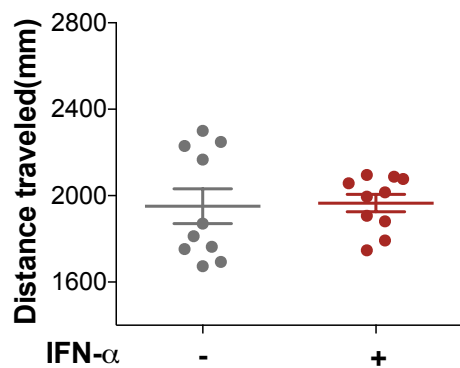**b.**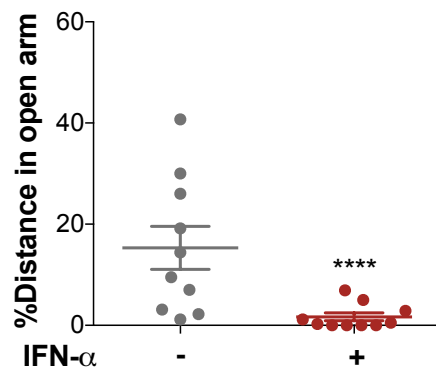**c.**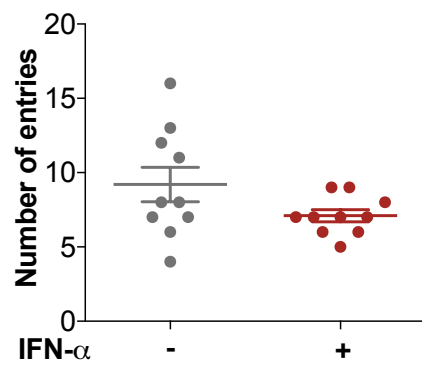**d.**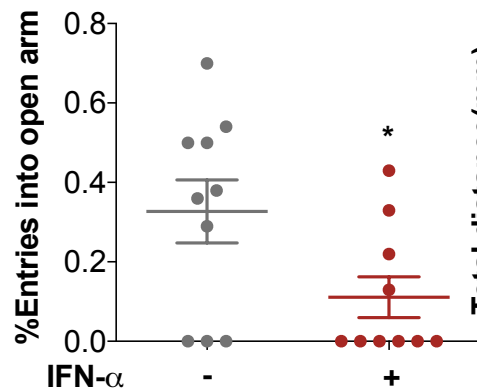**e.**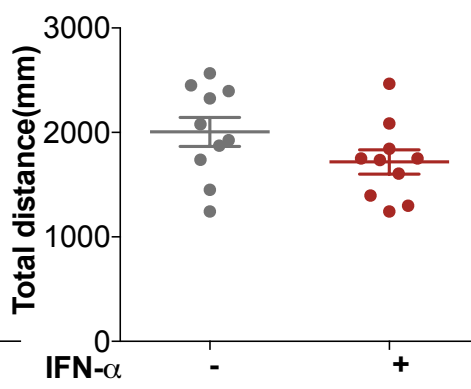**f.**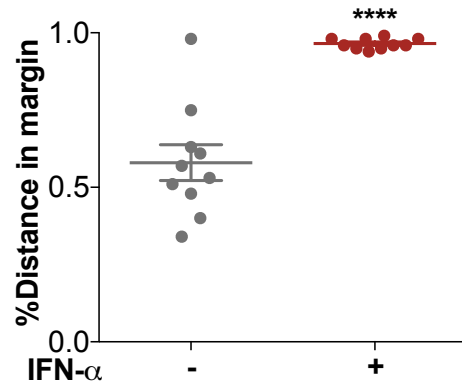**g.**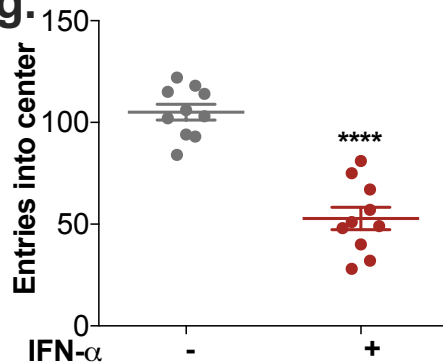

Supplement: Supplementary file 3 — Figure S3. Effect of IFN-α on anxiety-like phenotypes in NZB/NZW F1 mice, as assessed by the elevated plus-maze test. (a) Total distance travelled in the elevated plus-maze. (b) Percentage of distance travelled in the open arm of the elevated plus-maze. (c) Total number of entries into the arms of the elevated plus-maze. (d) Percentage of entries into the open arms. (e) Total distance travelled in the open field arena. (f) Percentage of distance travelled in the margin area in the open field test. (g) Number of entries into the centre of the open field. Number of animals per group =10. *P < 0.05, **P < 0.01, ***P < 0.005, ****P < 0.001 vs. Adv-ctrl treated group, unpaired t test. (PDF 44 kb) [file 13075_2019_1985_MOESM3_ESM.pdf]

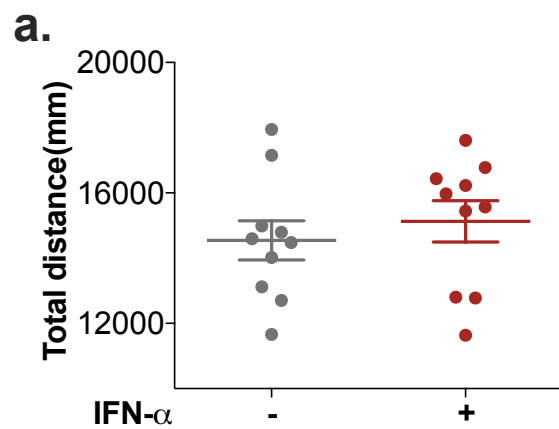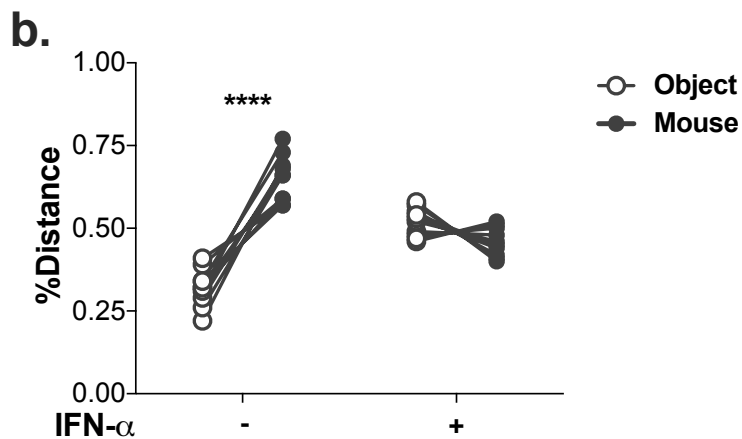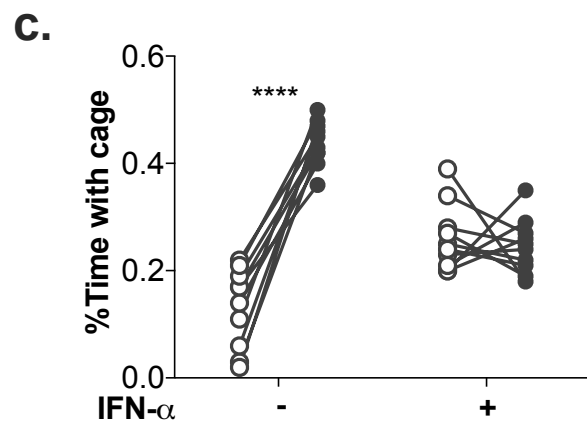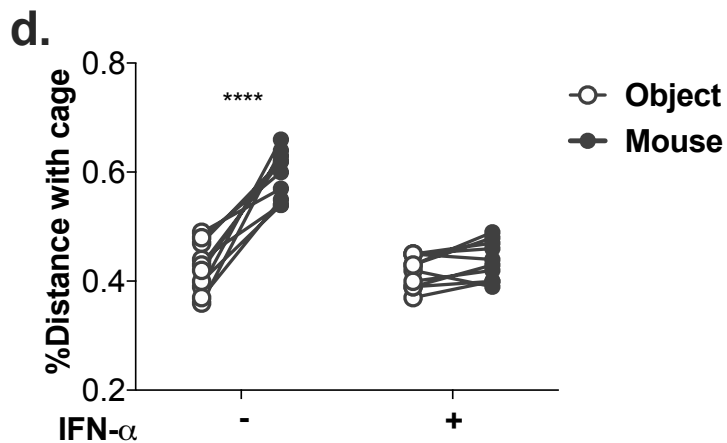

Supplement: Supplementary file 4 — Figure S4. Effect of IFN-α on sociability in NZB/NZW F1 mice, as assessed in the three-chamber social interaction test. (a) Total distance travelled in all of the three chambers. (b) Percentage of distance travelled in the two chambers. Percentage of time spent (c) and distance travelled (d) around the two cages. Number of animals per group =10. *P < 0.05, **P < 0.01, ***P < 0.005, ****P < 0.001 vs. Adv-ctrl treated group, unpaired t test (a), 2-way ANOVA with Sidak’s test (b, c, d). (PDF 55 kb) [file 13075_2019_1985_MOESM4_ESM.pdf]

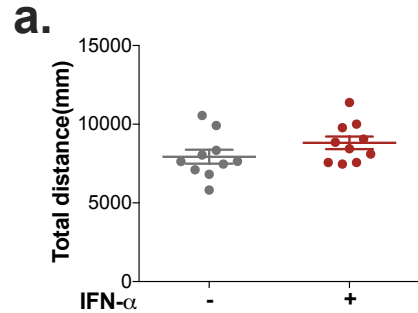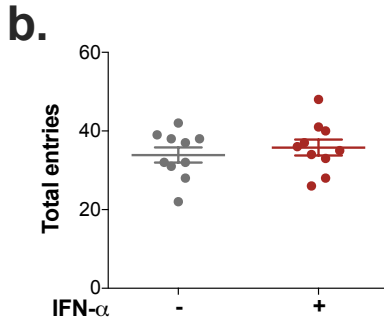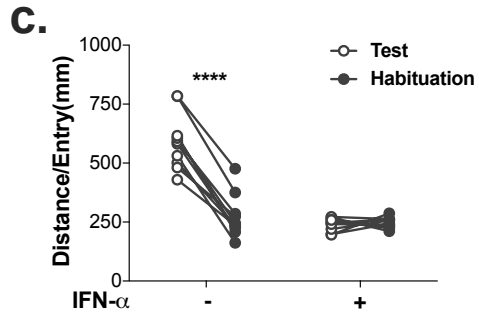

Supplement: Supplementary file 5 — Figure S5. Effect of IFN-α on sociability in NZB/NZW F1 mice, as assessed by the novelty Y-maze task. (a) Total distance travelled. (b) Total number of arm entries. (c) Distance travelled in the arms per entry. Number of animals per group =10. *P < 0.05, **P < 0.01, ***P < 0.005, ****P < 0.001 vs. Adv-ctrl treated group, unpaired t test (a, b), 2-way ANOVA with Sidak’s test (c). (PDF 35 kb) [file 13075_2019_1985_MOESM5_ESM.pdf]
